# Longitudinal metabolomics of increasing body-mass index and waist-hip ratio reveals two dynamic patterns of obesity pandemic

**DOI:** 10.1038/s41366-023-01281-w

**Published:** 2023-02-23

**Authors:** Ville-Petteri Mäkinen, Johannes Kettunen, Terho Lehtimäki, Mika Kähönen, Jorma Viikari, Markus Perola, Veikko Salomaa, Marjo-Riitta Järvelin, Olli T. Raitakari, Mika Ala-Korpela

**Affiliations:** 1 Systems Epidemiology, Faculty of Medicine, University of Oulu, Oulu, Finland; 2 Research Unit of Population Health, Faculty of Medicine, University of Oulu, Oulu, Finland; 3 Computational and Systems Biology Program, Precision Medicine Theme, South Australian Health and Medical Research Institute, Adelaide, SA Australia; 4 Australian Centre for Precision Health, University of South Australia, Adelaide, SA Australia; 5 Biocenter Oulu, Oulu, Finland; 6 Department of Health and Welfare, Finnish Institute for Health and Welfare, Helsinki, Finland; 7 Department of Clinical Chemistry, Fimlab Laboratories, and Finnish Cardiovascular Research Center Tampere, Faculty of Medicine and Health Technology, Tampere University, Tampere, Finland; 8 Department of Clinical Physiology, Tampere University Hospital, and Finnish Cardiovascular Research Center Tampere, Faculty of Medicine and Health Technology, Tampere University, Tampere, Finland; 9 Department of Medicine, University of Turku, Turku, Finland; 10 Division of Medicine, Turku University Hospital, Turku, Finland; 11 Institute for Molecular Medicine (FIMM), University of Helsinki, Helsinki, Finland; 12 Estonian Genome Center, University of Tartu, Tartu, Estonia; 13 Unit of Primary Health Care, Oulu University Hospital, OYS, Oulu, Finland; 14 Department of Epidemiology and Biostatistics, MRC-PHE Centre for Environment and Health, School of Public Health, Imperial College London, London, UK; 15 Department of Life Sciences, College of Health and Life Sciences, Brunel University London, London, UK; 16 Research Centre of Applied and Preventive Cardiovascular Medicine, University of Turku, Turku, Finland; 17 Department of Clinical Physiology and Nuclear Medicine, Turku University Hospital, Turku, Finland; 18 Centre for Population Health Research, University of Turku and Turku University Hospital, Turku, Finland; 19 NMR Metabolomics Laboratory, School of Pharmacy, University of Eastern Finland, Kuopio, Finland

**Keywords:** Ageing, Metabolism

## Abstract

**Background/Objective:**

This observational study dissects the complex temporal associations between body-mass index (BMI), waist-hip ratio (WHR) and circulating metabolomics using a combination of longitudinal and cross-sectional population-based datasets and new systems epidemiology tools.

**Subjects/Methods:**

Firstly, a data-driven subgrouping algorithm was employed to simplify high-dimensional metabolic profiling data into a single categorical variable: a self-organizing map (SOM) was created from 174 metabolic measures from cross-sectional surveys (FINRISK, *n* = 9708, ages 25–74) and a birth cohort (NFBC1966, *n* = 3117, age 31 at baseline, age 46 at follow-up) and an expert committee defined four subgroups of individuals based on visual inspection of the SOM. Secondly, the subgroups were compared regarding BMI and WHR trajectories in an independent longitudinal dataset: participants of the Young Finns Study (YFS, *n* = 1286, ages 24–39 at baseline, 10 years follow-up, three visits) were categorized into the four subgroups and subgroup-specific age-dependent trajectories of BMI, WHR and metabolic measures were modelled by linear regression.

**Results:**

The four subgroups were characterised at age 39 by high BMI, WHR and dyslipidemia (designated TG-rich); low BMI, WHR and favourable lipids (TG-poor); low lipids in general (Low lipid) and high low-density-lipoprotein cholesterol (High LDL-C). Trajectory modelling of the YFS dataset revealed a dynamic BMI divergence pattern: despite overlapping starting points at age 24, the subgroups diverged in BMI, fasting insulin (three-fold difference at age 49 between TG-rich and TG-poor) and insulin-associated measures such as triglyceride-cholesterol ratio. Trajectories also revealed a WHR progression pattern: despite different starting points at the age of 24 in WHR, LDL-C and cholesterol-associated measures, all subgroups exhibited similar rates of change in these measures, i.e. WHR progression was uniform regardless of the cross-sectional metabolic profile.

**Conclusions:**

Age-associated weight variation in adults between 24 and 49 manifests as temporal divergence in BMI and uniform progression of WHR across metabolic health strata.

## Introduction

We recently described how age-associated changes in body-mass index (BMI) are strongly associated with concurrent changes in a wide selection of metabolic measures over 15 years of follow-up in a large-scale population sample [1]. Counter-intuitively, we observed an increase in waist-hip ratio (WHR) even in the subset of individuals who lost weight during follow-up, which suggests that the trajectories of BMI and WHR may have complex hitherto unappreciated temporal relationships with systemic metabolism. Obesity trajectories have been described before [2], and they are associated with adverse outcomes [3], however, temporal differences between BMI and WHR are rarely investigated from a systems perspective. This is in contrast to other fields, such as developmental biology and precision cancer medicine, where modern data science methods including time-dependent clustering of high-dimensional omics datasets are now standard practice to gain novel insight into complex phenomena [4]. We have previously adapted the systems paradigm to cross-sectional and prospective epidemiological datasets to capture statistical patterns that would have been difficult to detect with traditional methods [5–7]. In this study, we adapt systems thinking from molecular biology to the epidemiology of obesity in a longitudinal setting. Our specific aim is to dissect temporal associations between systemic metabolism (measured by serum metabolomics) and the two most popular indicators of obesity (BMI and WHR) during an important period in adulthood that precedes the exponential rise in disease burden later in life.

The scientific value of metabolomics has been demonstrated in cardiovascular medicine [8–10] and in genetics [11], and the metabolome may predict cardiometabolic diseases [12, 13]. Yet the added data dimensionality can cause problems for traditional biostatistics. In response to the technical challenges, we and others have introduced data-driven subgrouping to gain deeper insight into complex biomedical phenomena [5, 14, 15]. In particular, the self-organizing map (SOM) methodology we developed and have been using for 15 years is uniquely designed for population-based human studies [5]. The SOM has been used before in prospective studies of clinical end-points [6, 7], but here we combine it with longitudinal biochemical data for the first time.

Previous subgrouping studies have stratified large population cohorts according to cross-sectional biochemical data [7, 15, 16], or by using cross-sectional age differences as proxies for longitudinal trajectories [17]. However, there are fewer examples that have defined subgroups regarding actual longitudinal data, despite the stronger evidence longitudinal analyses provide [18–22]. Dayimu et al. combined cross-sectional age spread with serial measurements of clinical lipids in 9726 participants and defined three lipid profile trajectories from 20 to 60 years of age (U-shape, progressing and an inverse U-shape [23]). Importantly, the authors concluded that managing lipid trajectories before the age of 42 may be crucial to effective disease prevention. In a study by Elovainio et al., subgroups of childhood lipid trajectories were compared against depressive symptoms as adults, and the authors found an increase in depression risk for a steeply increasing triglyceride trajectory [24]. These examples demonstrate why it is useful to describe longitudinal trajectories of metabolic measures as the bridge between early life predictors and late life disease burden in populations with high obesity rates [25].

For the current study, we collected a total of 174 metabolic measures (clinical biomarkers and NMR metabolomics) from a Finnish longitudinal dataset (YFS, *n* = 1286, ages 24–39 at baseline, three visits between 2001 and 2011) with supporting data from other population surveys (*n* = 12,825). These unique population resources enabled us to apply the SOM framework in a study design that separated subgroup construction from the evaluation of temporal obesity trends and metabolic trajectories (robust statistical conclusions are essential for ageing studies [1, 18, 22]). Importantly, the subgroup modelling opened an opportunity to investigate longitudinal BMI and WHR change – two crucial population and clinical obesity metrics – in relation to the broader patterns of systemic metabolism. Our results provide new previously unattainable insight into how BMI and WHR are associated with the metabolic transition from young adulthood at age 24 up to mid-life at 49 within a real-world human population.

## Materials and methods

### Cardiovascular risk in Young Finns Study (YFS)

**Fig. 1:**
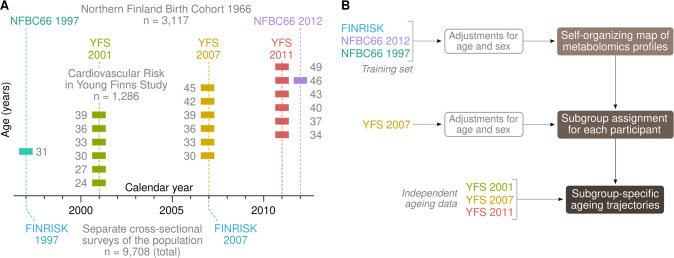
Description of datasets and study design. **A** Illustration of the age structure of the datasets. The coloured blocks indicate the specific ages the participants were at the time of blood collection. The age range for the FINRISK surveys was between 25 and 74 years. **B** Statistical study design.

The Cardiovascular Risk in Young Finns Study (YFS) is a population based prospective cohort study [26]. It was conducted at five medical schools in Finland (Turku, Helsinki, Kuopio, Tampere and Oulu), with the aim of studying the levels of cardiovascular risk factors in children and adolescents in different parts of the country. The baseline study in 1980 included 3596 children and adolescents aged between 3 and 18 years. Results from clinical examination and fasting samples were used in the present study. Metabolomics and clinical assays were available from three visits in 2001 (1239 women and 1007 men), 2007 (1186 women and 974 men) and 2011 (1112 women and 927 men). A visual overview of the dataset structure is shown in Fig. 1A.

### Northern Finland Birth Cohort 1966 (NFBC1966)

The NFBC1966 is a longitudinal birth cohort established to study factors affecting preterm birth and consequent morbidity in the two northernmost provinces of Finland, Oulu and Lapland [27]. The NFBC1966 includes 12,231 births (12,058 alive) covering 96% of all eligible births in this region during January-December 1966. Data collections in 1997 (at age of 31) and 2012 (age 46) including clinical examination and fasting serum sampling was used in the present study. Metabolomics and clinical assays were available from the 31-year (2962 women and 2749 men) and 46-year visits (3237 women and 2549 men).

### FINRISK

FINRISK surveys are cross-sectional, population-based studies conducted every five years since 1972 to monitor the risk of chronic diseases [28]. For each survey, a representative random sample between the ages 25 and 74 was selected from five regions in Finland. The current study included eligible participants from FINRISK surveys conducted in year 1997 and 2007. Data collection including clinical examination and serum samples were available for these two surveys. Serum samples were stored at −70 °C. Samples were semi-fasting: participants were asked not to eat 4 h prior to giving blood. The median fasting time was 5 h (interquartile range 4–6 h). Metabolomics and clinical assays were available from 5304 participants (mean age 48 ± 13 years) in the 1997 survey and 4616 participants (mean age 52 ± 13 years) in the 2007 survey.

### Metabolomics and clinical biomarkers

The following data were available at each date of blood collection. A high-throughput nuclear magnetic resonance (NMR) spectroscopy metabolomics platform was used to quantify 164 lipid and metabolite measures from serum [29]. The platform applies a single experimental setup, which allows for simultaneous quantification of standard clinical lipids, 14 lipoprotein subclasses and individual lipids (triglycerides, phospholipids, free and esterified cholesterol) transported by these particles, multiple fatty acids, glucose and various glycolysis precursors, ketone bodies and amino acids in absolute concentration units. In addition, glucose, insulin, triglycerides, LDL cholesterol, HDL cholesterol and C-reactive protein were assessed by standard clinical assays. Of note, we also checked the Homeostatic Model Assessment of Insulin Resistance (HOMA-IR) but it provided no extra information compared to insulin alone (Spearman R = 0.99). BMI (weight divided by height squared), WHR (midpoint between the lowest ribs and the top of the iliac crest divided by the widest horizontal section of buttocks), systolic and diastolic blood pressure were also included. The metabolic data used in this study (174 measures in total) are the same as in a previous publication that introduced new methods for sample quality control and correction for batch effects [1].

### Metabolic subgrouping

A self-organizing map (SOM) was constructed based on the FINRISK cross-sectional data and the NFBC66 data [5, 30]. The input variables included 174 quantitative metabolic traits that were available in all cohorts [1]. The data were adjusted for age and sex and standardized with the R function nroPreprocess(method = ”standard”) that calculates empirical z-scores but with protection from skewed distributions and outliers [5]. Each cohort was pre-processed separately to mitigate batch effects. Before training, collinear inputs were merged using an agglomerative network algorithm [31] which resulted in a final set of 53 non-redundant input features. To ensure temporal consistency with the 2007 YFS visit (mean age 39), the NFBC1966 data were interpolated to 39 years of age by the formula 0.47×NFBC1966_31yrs_ + 0.53×NFBC1966_46yrs_ before training the map. The SOM was fitted to the combined dataset of FINRISK surveys and the interpolated NFBC1966 visit. After training, the YFS participants were placed on the SOM according to the 2007 visit. Neither the 2001 nor the 2011 YFS visit was used for determining the positions of individuals on the map and are thus considered independent time points (Fig. 1B). Furthermore, a human committee determined biologically relevant subgroup boundaries on the map according to the cross-sectional visual patterns in the training data before longitudinal data were accessed. This enabled us to calculate meaningful *P*-values for observed longitudinal patterns in the YFS cohort from the 2001 to the 2011 visit.

### Temporal trajectories

Temporal trajectories that combined cross-sectional and longitudinal data in the YFS were modelled by linear regression. We tested quadratic and cubic models, however, the results were not substantially different or suffered from instability (data not shown). Each model included chronological age as the regressor, birth year as a covariate, and the quantitative metabolic measure as the dependent variable. The metabolic measure was standardized as implemented in the Numero function nroPreprocess(method = ”standard”), which log transforms skewed data, truncates extreme outliers and scales by standard deviation [5]. Age unit was set to one decade. Model fit was confirmed by visual inspection of residual plots. Confidence intervals for the regressor coefficient β_age_ were estimated by bootstrapping. In addition, analysis of variance (ANOVA) was conducted for the coefficients by permutation analysis. Analogous ANOVA was calculated for unadjusted subgroup means. Visual trajectories to depict the regression models were created by setting the confounders to zero and plotting the model outputs for the ages between 24 and 49.

### Multiple testing

Principal component analysis (PCA) of the biochemical data revealed that the first 48 PCA components explained 99% of the total variance when all data were pooled. These results were compatible with earlier work [32]. For consistency, we set the multiple testing threshold at the more conservative *P* < 0.0006 to match the previous paper (equivalent to Bonferroni adjustment for 83 independent tests at 5% type 1 error rate). All statistical analyses were conducted in the R environment version 3.6 (https://www.R-project.org). All *P*-values are two-sided unless otherwise indicated.

## Results

**Fig. 2:**
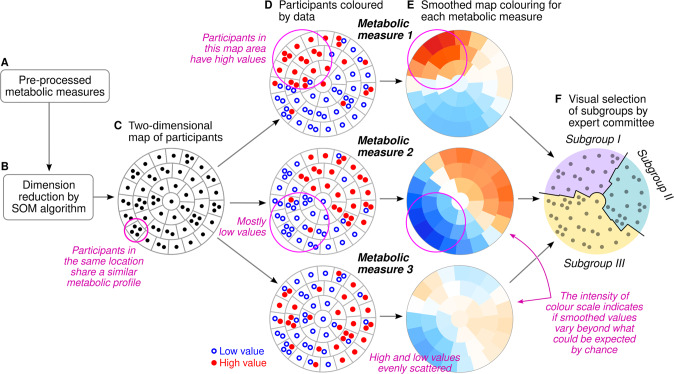
Schematic presentation of the self-organizing map (SOM) framework for creating data-assisted multi-variable subgroups. **A**, **B** Unsupervised clustering analysis. **C**–**E** Main concept of the data mapping. **F** Final classification step that produces a category label for each participant. The last step of assigning subgroups is done exclusively by human observers.

To explore the structure of the high-dimensional metabolic data, we described and summarized the variation of 174 metabolic measures using the self-organizing map (SOM). In the schematic example, each participant is represented by their individual pre-processed metabolic profile (Fig. 2A). The Kohonen algorithm [30] is applied to project the high-dimensional input data onto the vertical and horizontal coordinates (Fig. 2B). On the resulting scatter plot, proximity between two participants means that their full multivariable input data are similar as well (Fig. 2C). However, scatter plots are cumbersome for large datasets and difficult to interpret in the absence of distinct clusters. The SOM circumvents these challenges by dividing the plot area into districts. To show statistical patterns, each district is colored according to the average value of a single biomarker or, in the case of morbidity, the local prevalence or incidence of a disease (Fig. 2D, E). The connection between proximity on the canvas and similarity of full profile works the same way on the SOM as it does on a scatter plot. Therefore, selecting a region on the SOM is the same as selecting a subgroup of individuals with mutually similar profiles of input data (Fig. 2F).

Regarding the technical details of the SOM, we highlight extensive supplementary documents in four earlier papers that introduce the basic mathematical concepts and discuss the differences between textbook examples of clustered data and real-world population-like datasets [5, 31, 33, 34]. Practical documentation is also available in the vignette of the Numero R package (URL: https://cran.r-project.org/web/packages/Numero/vignettes/intro.html).

### Designation of metabolic subgroups based on cross-sectional metabolic features

**Fig. 3:**
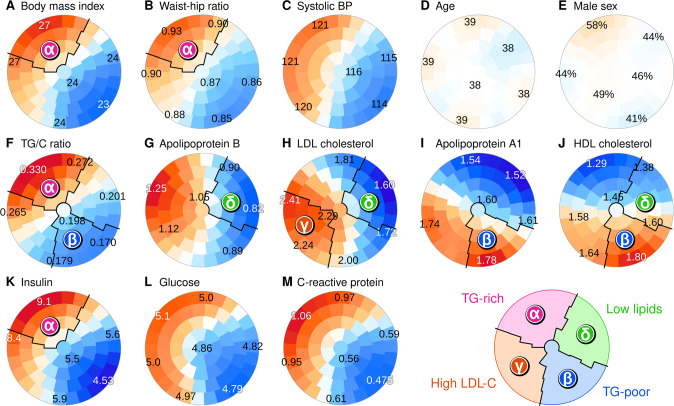
Metabolic subgrouping derived from the self-organizing map (SOM) of clinical and biochemical variables. **A**–**M** Selected map colorings that were especially investigated by the expert committee who drew the boundaries. The SOM was constructed from the FINRISK survey data and the Northern Finland Birth Cohort 1966 (adjusted for age and sex, details in Methods). The colorings were created based on the participants of the Cardiovascular Risk in Young Finns study who were placed on the map according to the data from 2007. Each plot represents the same map and the district colors indicate standardized differences from the population mean (red for higher and blue for lower values, color intensity indicates statistical robustness). Exact district means are written on selected locations. Colorings for all variables are available in Supplement 1.

A self-organizing map (SOM) was fitted to the FINRISK and NFBC66 data and adjusted for age and sex to further elucidate the relationship between cross-sectional metabolic diversity and longitudinal change (technical details in Methods). To summarize the multivariable profiles, we divided the SOM into four areas and assigned the participants located in each area to separate subgroups. In Fig. 3, we highlight the key features that the author committee used for deciding the subgroup boundaries (with priority given for features that would be widely available in other cohorts and in clinical practice), however, we considered all metabolic measures during the decision-making process (Supplement 1).

We first focused on the area of the map that was characterized by overweight (Fig. 3A, B). After examining patterns of all metabolic measures, we concluded that a high ratio of triglycerides to cholesterol is a biochemical feature with appealing properties: it is based on routine clinical biomarkers and differentiates the obese segment of the population accurately. Consequently, we designated the upper-left sector as the TG-rich subgroup (note also high insulin in Fig. 3K). We then applied the same thinking to the lower-right area of the map with the lowest body mass (Fig. 3F, designated as the TG-poor subgroup, note also HDL-related patterns in Fig. 3I, J). Of the two remaining areas, the lower left sector was characterized by high LDL cholesterol among others (Fig. 3H), and we designated it the high LDL-C subgroup given the important role LDL plays in the etiology of cardiovascular disease. The upper-right sector included individuals who had low circulating levels of all lipids (e.g. cholesterol and triglycerides), thus we designated them as the low-lipid subgroup.

### Detailed characterization of metabolic subgroups

**Fig. 4:**
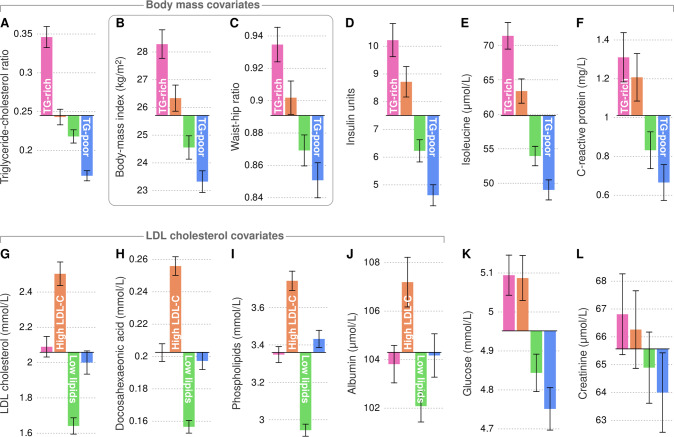
Mean values and 95% confidence intervals of selected measures for each metabolic subgroup. **A**–**F** A subset of measures that segregated the TG-rich from the TG-poor subgroup. **G**–**J** A subset of measures that segregated the high LDL-C from the low lipid subgroup. The statistics were calculated from the 2007 data of the Young Finns Study. Bar plots for all variables are available in Supplement 3.

**Fig. 5:**
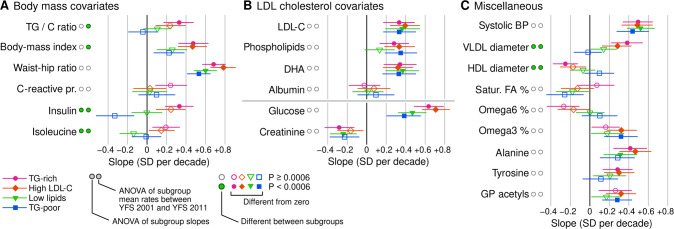
Temporal slopes of selected metabolic measures in the Cardiovascular Risk in Young Finns Study. **A** Variables that segregated TG-rich and TG-poor subgroups. **B** Variables that segregated high LDL cholesterol versus low lipid subgroups. **C** Other variables with previously known associations with metabolic health. The slopes and their 95% confidence intervals were estimated by linear regression across the 2001, 2007 and 2011 visits (see Methods for details). Full results are available in Supplements 4 and 5.

**Fig. 6:**
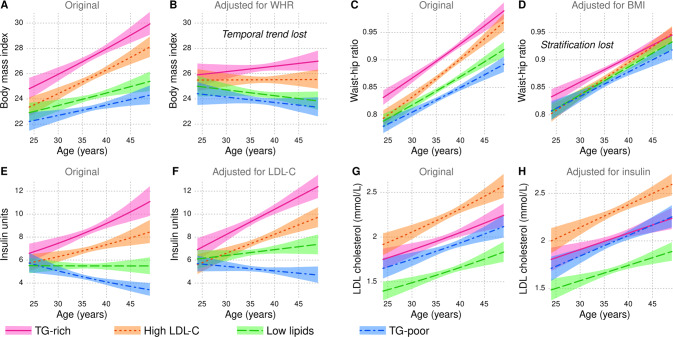
Visualization of longitudinal regression models of metabolic trajectories. **A**–**H** The models were fitted to the 2001, 2007 and 2011 data of the Cardiovascular Risk in Young Finns Study. The lines indicate the estimated output of the model with confounders set to zero and the colored areas indicate the 95% confidence interval. All metabolic trajectories are available in Supplement 6.

In the previous section, we created a data-assisted labelling of the participants into one of four metabolic subgroups. In this section, we characterize the two most important underlying cross-sectional patterns that differentiate the subgroup profiles (Fig. 4). In the next section, we present new data that shows how metabolic measures can follow a consistent cross-sectional pattern yet exhibit unexpected longitudinal behaviour (Fig. 5). Lastly, we present trajectory models that combine both cross-sectional stratification and longitudinal divergence to elucidate the dynamic relationship between BMI, WHR and clinically established biomarkers of metabolic health (Fig. 6).

Although we ended up with a lipid-focused subgrouping, we emphasize that these patterns are clearly visible across most if not all of the metabolic measures due to extensive collinearity (the correlation matrix is visualized in Supplement 2). To avoid narrative clutter, we focused on a core set of biomarkers that capture the systemic changes well and are widely used in clinical medicine and epidemiological studies.

The cross-sectional differences between the subgroups regarding a selected set of metabolic measures are summarized in Fig. 4 and conventional bar charts are available in Supplement 3. BMI and WHR subgroup means were consistent across multiple metabolic measures: by definition, the TG-rich subgroup was the most obese and exhibited the highest insulin, isoleucine and C-reactive protein concentrations (Fig. 4A–F). On the other hand, the TG-rich and TG-poor subgroups did not differ substantially with respect to LDL cholesterol or collinear lipid measures such as docosahexeonoic acid and total phospholipids (Fig. 4G–I). Instead, those features segregated the high LDL-C subgroup from the low lipid subgroup. Glucose was a mix of the two main patterns with higher concentrations in the TG-rich and high LDL-C subgroups and lower concentrations in the TG-poor and low lipid subgroups (Fig. 4K, see also systolic and diastolic blood pressure in Supplement 4). Creatinine was not different between the subgroups (Fig. 4L).

### Comparison of longitudinal rates of change between metabolic subgroups

Linear regression models were fitted to the YFS data to elucidate the slope of temporal trajectories of the SOM-derived metabolic subgroups (technical details in Methods). The standardized slope was quantified by the coefficient for age in the regression model (β_age_ = rate of change in population standard deviations per decade). The full list of coefficients is available in Supplement 5 with a comprehensive visualization in Supplement 6.

Temporal slopes of triglyceride-cholesterol ratio and insulin were associated with the cross-sectional subgroup label while weak or negligible divergence was observed for BMI and WHR (Fig. 5A). Insulin, specifically, produced strong statistical signals both in terms of diverging slopes (*P* = 9.3 × 10^−^^12^) and unadjusted rates of change (*P* = 5.9 × 10^−^^11^) between subgroups; insulin slopes were substantially different between the TG-rich subgroup (β_age_ = +0.35, CI95:+0.20, +0.49) and the TG-poor subgroup (β_age_ = −0.32, CI95:−0.51, −0.14) and some difference was observable between the Low lipid (β_age_ = −0.0082, CI95: − 0.17, +0.14) and TG-poor subgroups. The connection between cross-sectional subgroups and longitudinal slopes was also visible in multiple VLDL and HDL measures (Fig. 5C and Supplement 6) and in ratios of fatty acid chains (Fig. 5C).

For most metabolic measures, cross-sectional stratification did not predict longitudinal divergence, including C-reactive protein that one would have expected to be divergent based on the cross-sectional pattern (Fig. 4F vs. 5A). The slopes for LDL-C overlapped statistically between the high LDL-C (β_age_ = +0.46, CI95: + 0.30, +0.62) and low lipid subgroups (β_age_ = +0.29, CI95: + 0.15, +0.43) despite a highly significant 1.5-fold difference in concentration (Fig. 4G vs. Fig.5B).

Although there was limited divergence between subgroups, the overall magnitudes of change differed between metabolic measures. For example, the standardized slope for WHR was 84% faster compared to BMI, indeed, WHR and glucose exhibited some of the fastest slopes of any measure (0.78 for WHR and 0.71 for glucose in the high LDL-C subgroup). Most lipid measures increased in all subgroups (e.g. LDL cholesterol, phospholipids and docosahexaenoic acid in Fig. 5B, see also various triglyceride measures in Supplement 6), but albumin was an example of a measure that was stratified cross-sectionally, but exhibited no longitudinal change (Fig. 4J vs. Fig. 5B). We observed a consistent negative slope for creatinine across all subgroups (Fig. 5B).

### Metabolic trajectories modelled from cross-sectional and longitudinal data

To elucidate the qualitatively different dynamics of BMI and WHR, we constructed trajectory models of the YFS dataset (details in Methods, full results in Supplement 7). Furthermore, we also adjusted BMI with WHR and vice versa to assess the overlap between them. As expected, both measures exhibited an increasing trend in all subgroups and were stratified already in the beginning of the follow-up period (Fig. 6A, C). However, much of the temporal trend could be explained by WHR alone, since subgroup-specific changes in BMI reverted towards zero if adjusted for WHR (Fig. 6B). Conversely, adjustment by BMI removed much of the stratification between subgroups while the temporal trend in WHR was preserved and highly consistent regardless of the cross-sectional metabolic profile (Fig. 6D).

In the TG-rich subgroup, estimated mean BMI increased from the overweight threshold of 25 kg/m^2^ to the obesity threshold of 30 kg/m^2^ during the 25-year time span – this means that by the sixth decade of life, more than half of these individuals are likely to become obese (Fig. 6A). During the same period, the models predicted that the majority of the TG-poor subgroup will remain within a healthy range of BMI. From a biomolecular perspective, the divergence was the most extreme with respect to insulin (see also triglyceride-cholesterol ratio in Supplement 7): all four subgroups overlapped in early twenties, however, there was an estimated three-fold difference in insulin at the age of 49 between the TG-rich and the TG-poor subgroups (Fig. 6E).

Lastly, we compared the metabolic trajectories of insulin and LDL cholesterol as a minimal set of parsimonious biomarkers that can capture the broad strokes of metabolic ageing. Evidently, fasting insulin is well suited to capture individuals with rapidly deteriorating energy metabolism (Fig. 6E), while the trajectories of LDL cholesterol were stable even when the starting points were substantially different (parallel curves in Fig. 6G). From a statistical perspective, insulin and LDL cholesterol trajectories did not overlap to the same extent as WHR and BMI (adjusting one by the other did not alter the trajectories substantially in Fig. 6F, H).

## Discussion

We leveraged multiple population-based cohorts and data mining algorithms to investigate how ageing manifests in metabolic measures obtained from repeated collections of blood over a decade. To make it easier to interpret multivariable statistical patterns in the data, we summarized the metabolic profiles by four representative subgroups (TG-rich, TG-poor, High LDL-C and Low lipids). We observed a qualitative difference between BMI and WHR regarding their temporal associations with the four metabolic subgroups. BMI and particularly insulin exhibited a divergent pattern where initial subgroup differences were weak at age 24 but became more pronounced by the time the individuals were 49 years old (i.e. those who were overweight at the start also gained weight more rapidly). On the other hand, WHR and particularly LDL-C where substantially different between the subgroups at 24 and the stratification remained almost unchanged up to 49 years (i.e. WHR increased at the same rate regardless of the starting point).

We characterised the population by four subgroups that we labelled according to established clinically available lipids (triglycerides and LDL cholesterol). These choices were made based on the vital importance the two main circulating lipids play in metabolic processes and cardiometabolic risk assessment in the clinics [6, 35–37], indeed, they are used as treatment targets for drugs aimed at reducing cardiovascular events [35, 38]. We and others have previously shown the association between triglyceride-rich metabolic profile and adverse outcomes [6], most recently for new-onset diabetes [39], and multiple age-associated diseases in the UK Biobank [7]. Here, we describe the dynamic context to these findings in an age range that precedes these types of late-onset diagnoses by multiple decades. This is important because addressing the clinical manifestations later in life is costly and difficult, whereas better understanding of how systemic metabolism gradually deteriorates in diverse human populations will give us better focus on how to improve the nature and timing of public health interventions.

The subgroups highlight one of the main aspects of the public health challenge of obesity. The gradient from TG-rich to TG-poor is temporally persistent, which supports the idea of biological resistance against an individual’s deviation from a pre-determined trajectory if the (obesogenic) environment stays the same, even if interrupted by episodes of dieting [40, 41]. From a scientific perspective, new time-series studies to elucidate the genetic and epigenetic programming and its interaction with environmental exposures across decades of human life would be necessary to discover effective ways to permanently re-adjust the programming.

The central role of obesity as a determinant of the metabolic profile is appealing from a causal perspective [42] and excess weight at a young age is a precursor to metabolic diseases later in life [3, 43]. We cannot confirm causality in this study, but our observations suggest that insulin action (proxied by circulating insulin concentration) is a good candidate for a mechanistic pivot that could explain most of the lipoprotein lipid, amino acid and other metabolic divergence between the subgroups [32, 44, 45]. When considering both the starting point and the trends, the modelling in Fig. 6 indicates that most people in their early twenties are remarkably similar with respect to fasting insulin, but highly stratified by the sixth decade of life. If we accept the hypothesis that insulin is the primary driver, preventing hyperinsulinemia would be an important public health goal. Under this scenario, our observations on the temporal dynamics of obesity provide important systems-based insight to guide further work. For example, maintaining the starting BMI at around 25 kg/m^2^ might be insufficient to stabilize insulin in individuals with the TG-rich metabolic profile, whereas accumulation of extra kilos on the waist for someone with a TG-poor profile would be less of a concern. This proposition is best supported by the specific observation that even when the waist-hip ratio in the TG-poor subgroup at age 48 *surpassed* the TG-rich at age 33 (Fig. 6C), there was no increase in insulin (Fig. 6E). Further studies are warranted to test if the TG-rich group will benefit from life-style or pharmacological interventions earlier in life compared to others.

Our longitudinal observations of the TG-poor subgroup are relevant for the concept of healthy obesity [46]. Proponents of the concept argue that having a healthy metabolic profile is more important regarding clinical outcomes than focusing on weight [47] while the opposition points out that those who are obese convert to an adverse metabolic phenotype, given enough time [48].

This study cannot directly address the first argument since the participants are too young to have statistically meaningful rates of overt diseases. Nevertheless, we can confirm the existence of individuals with a stable insulin trajectory despite a widening waist (i.e. TG-poor), therefore we propose that focusing on the temporal correlation between adiposity and insulin could be a powerful and well-defined way to stratify the metabolic resilience of overweight individuals. This insulin-centric approach could also help resolve the main weakness of the healthy obesity concept, namely that there is no clear definition of what “healthy” actually means in this context [46].

We also agree with the counter argument: everyone in our study showed consistent deterioration in classical cardiovascular risk factors such as LDL cholesterol and blood pressure regardless of the BMI and WHR trajectories or starting points. Based on this, we speculate that a resilient metabolic phenotype against obesity would not protect against the cholesterol-driven or hemodynamic components of atherosclerosis etiology.

### Strengths and weaknesses

Two independent longitudinal cohorts and two independent cross-sectional surveys of the same ethnicity, socioeconomics and time period provide us with robust data and high statistical power, but these strengths also mean that the results may not generalize outside the Northern European context. Furthermore, the results apply to adults under the age of 50 and further studies are needed to establish explicit links to late-life phenomena or how diet, exercise and genetics may influence the ageing trajectories of metabolite concentrations [49–51]. In older age groups, medications that improve circulating lipoprotein lipids (e.g. statins), blood pressure (e.g. ACE inhibitors) and glucose metabolism (e.g. metformin) are common and cause substantial changes to metabolic profiles, however, the prescription rates of these drugs were low in the relatively young and healthy participants in this study. Furthermore, our previous analyses on the UK Biobank indicate that medications have a limited impact on the multi-variable profiles despite larger effects on specific metabolic measures [7].

The results from this study do not represent causal evidence. Subgrouping and clustering studies are always at risk of finding patterns where none exist, however, we employed rigorous statistical designs to prevent overfitting and our subgroups are fully compatible with previous studies of other cohorts [6, 7], which gives us confidence that the subgroups are biologically meaningful. It must be emphasized, however, that metabolic subgrouping is far from deterministic for an individual point of view despite the strong population-wide associations – this paradox is a well-known feature of the epidemiology of common cardiometabolic diseases [52, 53].

The availability of metabolic measures is always limited and selected due to the technical constraints of analytical platforms and due the nature of the biosamples themselves. In this study, our data is lipid-centric, which means that lipids as the defining subgroup indicators can get over-emphasized. On the other hand, we took steps to manage collinearities to get an agnostic fit of the SOM, yet the ratio of triglycerides to cholesterol still emerged as the prominent feature. As these two lipids (and lipoprotein particles more broadly) are true and tested indicators of cardiometabolic health, we maintain our subgroup definitions are biologically relevant and accurate.

### Additional information from metabolomics versus established risk factors

The subgroup labels were predictable at 53% accuracy (versus 25% for random labels) by the full suite of available metabolic data and at 52% accuracy by nine pre-selected easily available clinical biomarkers (BMI, WHR, systolic and diastolic blood pressure, triglycerides, cholesterol, LDL cholesterol, HDL cholesterol and glucose; please see Supplement 8). The additional information from metabolomics was negligible (*P* = 0.57), which means that practical applications of metabolic ageing are likely to be feasible based on a panel of conventional biomarkers.

## Conclusions

Multi-variate subgrouping of longitudinal metabolomics data revealed two temporal features of the obesity pandemic that were captured by the two most commonly used markers for excess adiposity, BMI and WHR. We interpret the stratification and divergence of BMI in adulthood as a modifiable health indicator that segregates low and high-risk cardiometabolic phenotypes by diverging insulin trajectories and associated changes in triglyceride-cholesterol ratio and VLDL-HDL balance. On the other hand, the general increase in WHR may represent a hard-wired decline in cardiometabolic health that affects everyone and features consistently increasing rates of classical heart disease risk factors such as LDL cholesterol and blood pressure.

## Supplementary information

SOM colorings of YFS

Correlations

Subgroup profiles

Subgroup profiles for all measures

Ageing slopes

Ageing slopes visualization

Subgroup trajectories

Subgroup prediction

## Data Availability

The datasets used in the current study are available from the cohorts through application process for researchers who meet the criteria for access to confidential data: https://thl.fi/en/web/thl-biobank/for-researchers/apply (FINRISK 1997 cohorts), https://www.oulu.fi/nfbc/ (NFBC1966), and http://youngfinnsstudy.utu.fi (YFS). Regarding the YFS data the Ethics committee has concluded that under applicable law, the data from this study cannot be stored in public repositories or otherwise made publicly available. The data controller may permit access on case-by-case basis for scientific research, not however to individual participant level data, but aggregated statistical data, which cannot be traced back to the individual participants’ data.
