# Supplementary figures and images for: Longitudinal metabolomics of increasing body-mass index and waist-hip ratio reveals two dynamic patterns of obesity pandemic
Source: Int J Obes (Lond). 2023 Feb 23;47(6):453–62. doi: 10.1038/s41366-023-01281-w (PMC10212764; doi:10.1038/s41366-023-01281-w)

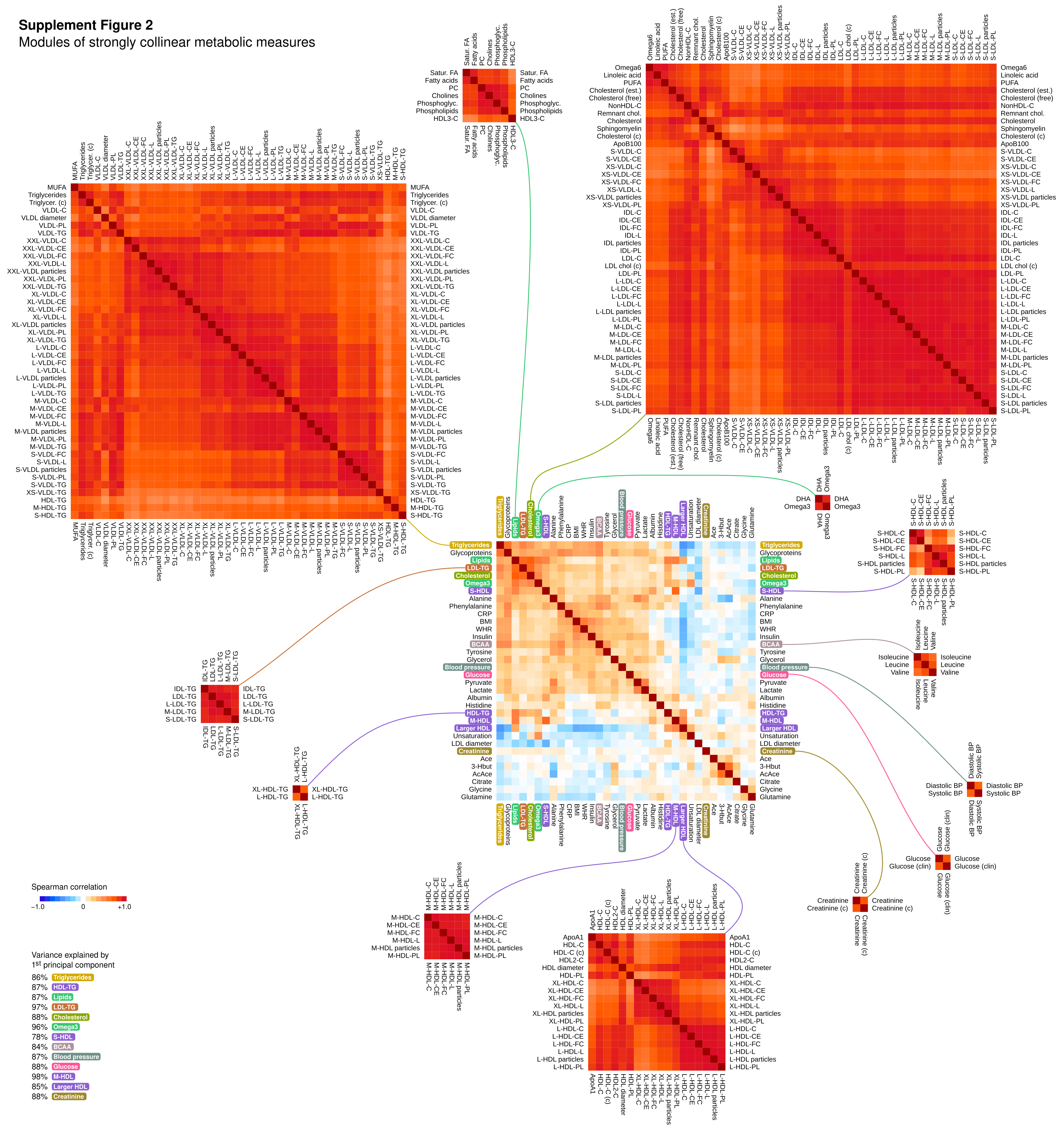

Supplement: Supplementary file 2 — Correlations [file 41366_2023_1281_MOESM2_ESM.png]

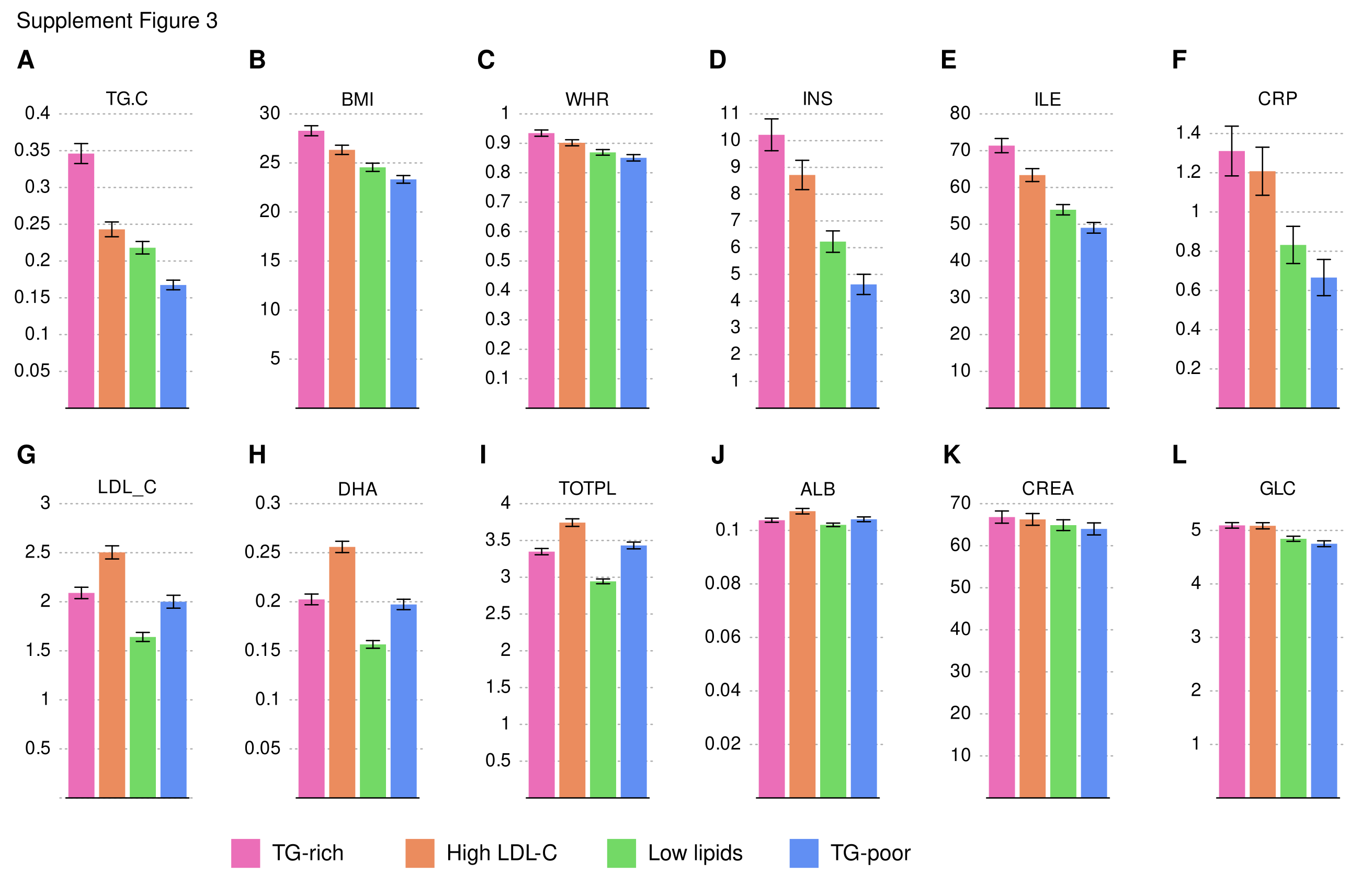

Supplement: Supplementary file 3 — Subgroup profiles [file 41366_2023_1281_MOESM3_ESM.png]

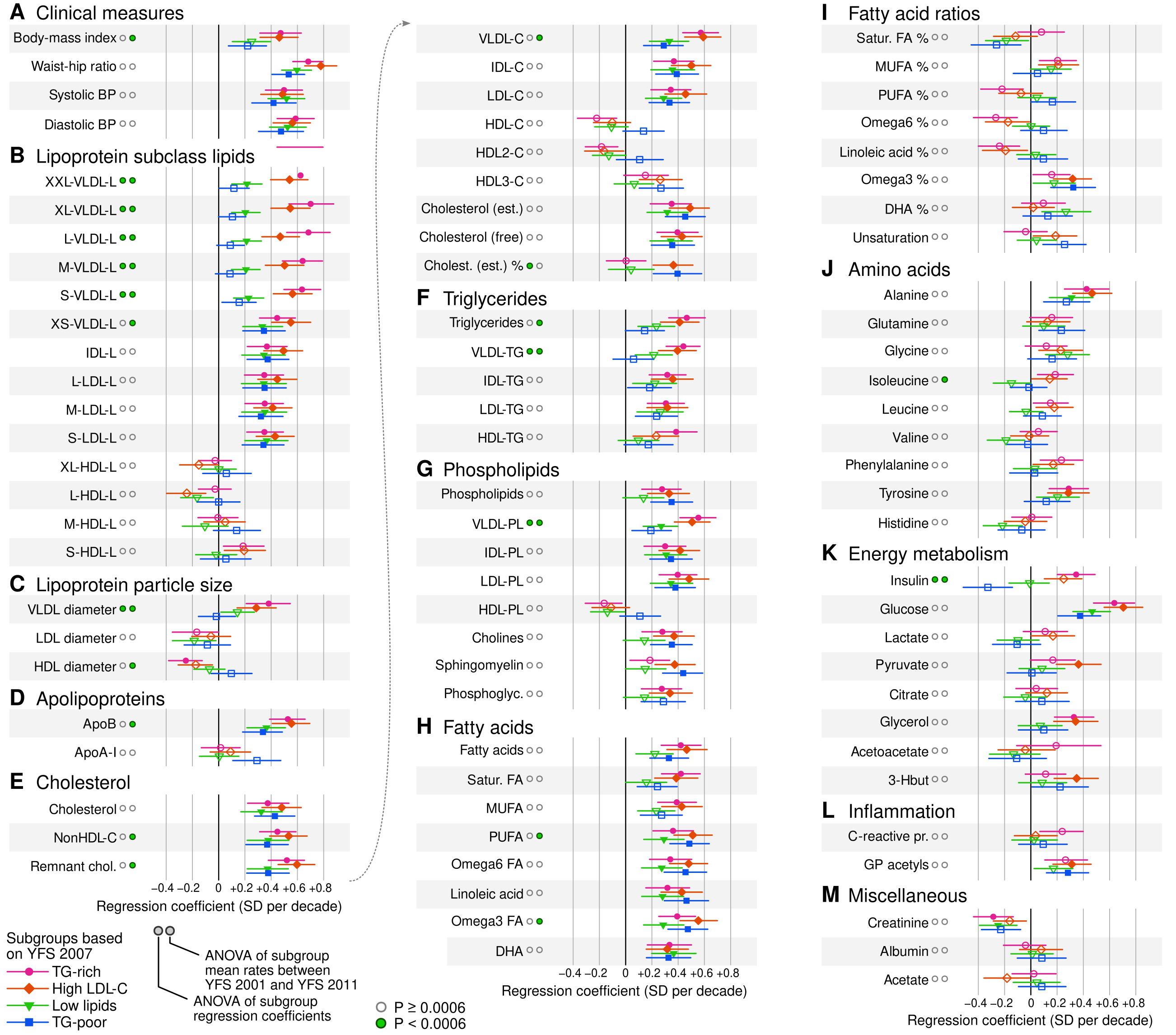

Supplement: Supplementary file 6 — Ageing slopes visualization [file 41366_2023_1281_MOESM6_ESM.png]
